# Replacing fossil oil with fresh oil – with what and for what?

**DOI:** 10.1002/ejlt.201100032

**Published:** 2011-07

**Authors:** Anders S Carlsson, Jenny Lindberg Yilmaz, Allan G Green, Sten Stymne, Per Hofvander

**Affiliations:** 1 Department of Plant Breeding and Biotechnology, Swedish University of Agricultural SciencesAlnarp, Sweden; 2 Scandinavian Biotechnology Research (ScanBiRes) ABAlnarp, Sweden; 3 CSIRO Plant IndustryCanberra, ACT, Australia

**Keywords:** Chemical feedstock, Novel oil accumulation, Oil crops, Specialty oils, Technical fatty acids, Vegetable oil

## Abstract

Industrial chemicals and materials are currently derived mainly from fossil-based raw materials, which are declining in availability, increasing in price and are a major source of undesirable greenhouse gas emissions. Plant oils have the potential to provide functionally equivalent, renewable and environmentally friendly replacements for these finite fossil-based raw materials, provided that their composition can be matched to end-use requirements, and that they can be produced on sufficient scale to meet current and growing industrial demands. Replacement of 40% of the fossil oil used in the chemical industry with renewable plant oils, whilst ensuring that growing demand for food oils is also met, will require a trebling of global plant oil production from current levels of around 139 MT to over 400 MT annually. Realisation of this potential will rely on application of plant biotechnology to (i) tailor plant oils to have high purity (preferably >90%) of single desirable fatty acids, (ii) introduce unusual fatty acids that have specialty end-use functionalities and (iii) increase plant oil production capacity by increased oil content in current oil crops, and conversion of other high biomass crops into oil accumulating crops. This review outlines recent progress and future challenges in each of these areas.

**Practical applications:** The research reviewed in this paper aims to develop metabolic engineering technologies to radically increase the yield and alter the fatty acid composition of plant oils and enable the development of new and more productive oil crops that can serve as renewable sources of industrial feedstocks currently provided by non-renewable and polluting fossil-based resources. As a result of recent and anticipated research developments we can expect to see significant enhancements in quality and productivity of oil crops over the coming decades. This should generate the technologies needed to support increasing plant oil production into the future, hopefully of sufficient magnitude to provide a major supply of renewable plant oils for the industrial economy without encroaching on the higher priority demand for food oils. Achievement of this goal will make a significant contribution to moving to a sustainable carbon-neutral industrial society with lower emissions of carbon dioxide to the atmosphere and reduced environmental impact as a result.

## Introduction

For the last 100 years fossil oil has been the cheapest raw material for energy and chemical production. Much of the materials and infrastructure of modern society has been built on the availability of this cheap source. This situation is now drastically and rapidly changing due to dwindling oil reserves in combination with increasing demand, with oil prices rising to up to 10 times those of 15 years ago. The previous low price of fossil oil provided little incentive to implement technologies that could improve energy efficiency, or replace fossil oils in industrial chemicals and materials production, even though substantial reductions in fossil oil consumption can be made easily and economically by applying existing technologies in these areas. However, recent decades have seen a rapid increase in industrialisation in the previous third world countries that are now approaching western countries in their life expectancy, living standards and energy consumption. Furthermore, global population growth, although predicted to level off in the latter half of this century, will still increase by 34% over the next 40 years [1]. Therefore, even if substantial energy efficiency measures can be implemented, alternative sources for energy and material will still need to be found to satisfy future demand levels that could reach twice that of current consumption of fossil oil, if the global development and standard of living expectations are to continue being met.

It is of course desirable to try to find alternatives that have substantially equivalent properties to fossil oil since much of the infrastructure needed is already in place, i.e. the combustion engines, power stations and the petrochemical industry. One obvious source would be fossil coal that can be converted to various hydrocarbons through chemical processes. Known coal reserves could probably provide society with its needs for an additional couple of hundred years. However, this is not a desirable option since it would mean unprecedented carbon dioxide emissions, escalating the already alarming global warming. Furthermore, the technologies for producing fuel and chemicals from coal are much more expensive than from fossil oil and natural gas.

Fossil oil and coal both originate from ancient biological material (predominantly from plant and algal sources) and represent a highly reduced form of the same biological material that is produced on the planet today. It is therefore logical to consider newly produced biological material as a replacement source for energy and carbon. Vegetable (plant) oils have a close resemblance to the chemical structures of fossil oil and can be processed to replace it in virtually any application. The present (2008/2009) annual world production of vegetable oil in agriculture is about 139 million metric tonnes (Mt) [2] – equivalent to only around 3% of the global fossil oil consumption – and the majority (about 80%) is used as food. Thus, there is no way that vegetable oil can make any significant contribution to replacing fossil oil for energy use (today about 4 billion tonnes (Gt) annually), even if the production was increased many times more.

It has recently been suggested that algal oil might replace a significant proportion of the fossil oil. Theoretical yield of biomass from microalgae is much higher than for terrestrial plants and figures exceeding 100 t dry mass/ha have been reported [3, 4]. If 50% of this was oil, it would give 50 t of oil/ha, about 10 times that produced by oil palm, the most productive terrestrial oil producing plant. However it remains to be proven that anywhere near such yields can be obtained on the extensive scale needed and one expert in the field has even called these claims absurd and bizarre [5]. Furthermore, even if such yields could be obtained, replacement of the fossil oil consumed today would require around 80 million ha of ponds to be constructed world-wide, controlled growth in these ponds achieved, and harvest and extraction facilities of hitherto unseen dimensions developed. The investment and running costs for such facilities are likely to make the extracted algae oil extremely expensive and the net energy output might be relatively small.

Considerable investments have been made into the development of the so-called second generation of biofuels using lignocellulose. The fuel might be obtained from fermentation (such as to ethanol) or by thermo-chemical conversion in the form of syngas and various hydrocarbons. It has been argued that the lignocellulose could be produced on marginal lands that are not in use for agriculture and therefore would not compete with food and feed production. However, these marginal lands are not suitable for agricultural production primarily due to their low biomass yield potential. Thus, the acreage would need to be many times the present agriculture acreage in order to make any significant contribution to replacing fossil oil for energy. Note that the global annual agricultural harvest (about 6 Gt) has an energy value of about one third that of the fossil oil consumed annually. It should also be noted that it has been argued that the most efficient use of biomass for transportation energy is not to produce ethanol or syngas from it, but to burn it to generate electricity in heat-power stations to fuel electrical vehicles [6].

Considering the above, we conclude that the infrastructure that was rational to use when cheap fossil oil was available will become highly uneconomic, although perhaps feasible, when using biofuels compared to other non-carbon based means of producing renewable energy, such as electricity from sun, wind, water and nuclear power.

Although it is highly unlikely that plants will play a significant role in replacing fossil oil for energy, the prospects of doing so for material and chemicals are much more favourable. Plant material, mainly in form of forest products, are already dominating over material made from fossil oils as sources of industrial materials. For example, the world annual consumption of paper and sawn wood (together about 650 Mt 2006) [7] is about 50% higher than the amount of fossil oil used by the chemical industry.

The chemical industry of 100 years ago was reliant on plant material as feedstock but the combination of availability of cheap fossil oil, the advancement of organic chemistry and increasing prices for plant material, led to a shift to a near total dependency on fossil oil as feedstock. Like the use of fossil oil for energy, the economics of the use of fossil oil in the chemical industry has been built on its low cost and wide availability. The cracking of fossil oil and building the desired products with advanced organic chemistry requires both large energy inputs and huge investments in infrastructure. The total value of the products and chemical made from the 10% of fossil oil used in this way is the same as that of all the fuels made from the other 90% [8]. Using plant products instead of fossil oil as the source for the material and chemicals could capture much of this added value if the plant molecules are optimised in planta for the end use and thereby minimising the downstream processing costs.

About 20% of the plant oils produced today is used in the oleochemical industry because for certain applications they are cheaper to use than fossil oil due to lower downstream processing costs. This has been the case for many oleochemicals even when the price difference between fossil oil and plant oil was considerably greater than today. Whereas the cost of plant oils 15 years ago was over five times higher than crude fossil oil, today they are approaching price parity. Thus, applications in which plant oils could not previously compete with fossil oil are now becoming economically viable.

Using plant oils in the chemical industry will not only enable replacement of fossil oil but will, at least equally importantly, enable substantial overall energy savings and generate added-value for agricultural products that cannot be captured by using them for energy production. We outline below research strategies for enabling the replacement of 40% of the fossil oil used in the chemical industry with plant oils within 20 years time by extrapolating on the present state of the art of science in the area. It should be pointed out that this is a science based exercise and does not take into account the petrochemical co-operative forces and other circumstances that might counteract this transformation into a bio-based chemical industry.

## Setting the scene: Replacing 40% of fossil oil with plant oils in chemical industry in 20 years time

The chemical industries use about 400 Mt of fossil oil annually in the production of many thousands of different chemicals and material. The two main uses are in manufacture of plastics and lubricants. It can be anticipated that within 20 years, the global demand from the chemical industry will increase to about 600 Mt to meet increased requirements coming from anticipated population increases and further economic development. Assuming the ability to produce the additional 240 Mt of plant oils required to replace 40% of this fossil oil, two important additional requirements will also need to be met. Firstly, the plant oil should be cheaper to use than fossil oil and provide at least the same final product performance. Secondly, the supply and price of the plant oil will need to have at least equivalent degree of security and predictability as fossil oil.

### Performance and price

In the applications where plant oils are today preferred over fossil oils in the chemical industry, it is because of their low downstream processing costs to the final product. The biggest technical market for plant oils today are detergents, mainly soaps, i.e. sodium salts of fatty acids, which are easily obtained by heating the oil with sodium hydroxide. Particularly useful in soap manufacturing are oils with shorter chain fatty acids such as lauric acid (12:0). Another substantial market for plant oils is in the production of erucamide, a slipping agent for plastic bags, made from erucic acid (22:1^Δ13^) oils. Lauric acid and erucic acid have global markets of about 1.4 and 0.1 billion US dollars, respectively [9, 10]. These are examples that show that the particular fatty acid structure is decisive for its economic viability as industrial feedstock. Both lauric and erucic acid are derived from plant oils (e.g. coconut and high-erucic rape) that are dominated by these acids (45–70% of all fatty acids).

Most oil crops contain five major fatty acids in their oil, which differ in their relative proportion depending on plant species; palmitic acid (16:0), stearic acid (18:0), oleic acid (18:1^Δ9^), linoleic acid (18:2^Δ9,12^) and α-linolenic acid (ALA, 18:3^Δ9,12,15^). Each of these fatty acids have their particular, but limited, use in chemical industry, but need to be purified from the mixture of other fatty acids in the oil, adding extra costs to the process.

In addition to these fatty acids, there are a huge number of more ‘exotic’ fatty acids occurring in seed oils from wild species that have a great potential in the chemical industry due to presence of functional groups such as double bonds at unusual positions, conjugated double bonds, triple bonds, hydroxy groups, epoxy groups and cyclopropenoid rings [11, 12]. Although some of these fatty acids can be present in very high amounts in the seed oils from wild species, these plants will have a very poor seed yield and therefore the particular fatty acids cannot be produced at a competitive cost.

Plant oils predominantly consist of TAG molecules, where three fatty acids are each esterified to one of the three hydroxy groups of a glycerol molecule. An interesting and unique exception is jojoba (*Simmondsia chinensis*) which accumulates seed oil in form of wax esters, esters between a fatty acid and a fatty alcohol. Wax esters are excellent lubricants due to their high stability under high temperature and pressure. However, the jojoba plant is a low yielding desert shrub and the jojoba oil is too expensive to be competitive for the use as industrial lubricants.

In addition to seed oils, plants produce a variety of extracellular lipids in form of suberin, cutin and aerial wax layers. These lipids contain a range of acyl chains with potential value in chemical industry, including dicarboxylic acids, omega (ω)-hydroxylated fatty acids, fatty alcohols, alkanes and wax esters [13, 14]. As a percentage of total biomass, these lipids only constitute <1% with some exceptions, such as the Carnauba palm (*Copernicia cerifera)*, whose thick high melting point leaf wax layer is harvested for use as a polishing agent [15].

In order to make plant oils more competitive in the chemical industry, in planta optimisation of their composition will be necessary. For certain oil qualities and in certain oil crops, this can be done using traditional mutation breeding technologies. However, in most cases it requires the use of genetic engineering. The prime goal of such genetic engineering is to obtain as high proportion as possible of the desired fatty acid in the oil. This fatty acid could be a fatty acid that already exists in the oil or it might be a fatty acid that is novel for the species. In the first section of this review, we will give examples of what already has been achieved towards this goal and point out bottlenecks experienced as well as possible routes to overcome these bottlenecks.

### Predictable and reliable supply

**Figure 1:**
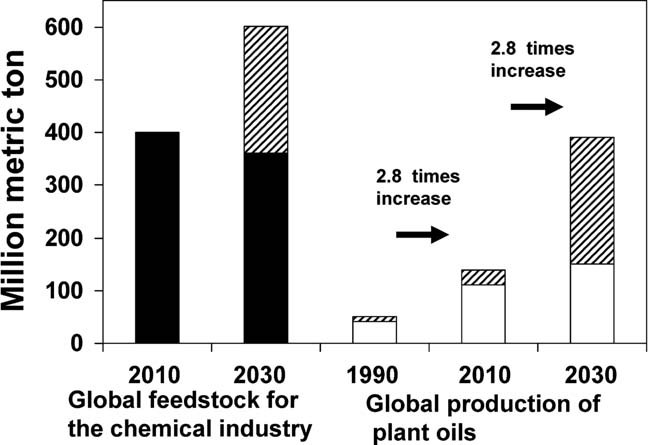
Actual and forecasted global feedstock needs for the chemical industry and plant oil production required for replacement of 40% of feedstock for chemical industry by 2030. Values at 2030 have been forecasted assuming yearly increases in the amount of feedstock needed by the chemical industry and the amount of oil needed for food of about 2 and 1.5%, respectively. Black bar areas indicate fossil oil, hatched areas indicate plant oils for chemical industry and open bar areas indicate plant oils for food and feed.

In order to replace 40% of fossil oil in the chemical industry with plant oils, we have to triple the plant oil production compared to today's 139 Mt of which about 111 Mt are used for food. In 20 years time, it is likely that around 150 Mt of plant oils are needed for food purposes due to the population growth and increasing affluence. To also provide the additional 240 Mt plant oil required to meet 40% of chemical industry demand without impacting food oil demand, would require a trebling of total plant oil production from 139 to 390 Mt (Fig. 1). This is the same rate of increase as achieved over the past 20 years, which has seen approximately 2.8-fold increase (from 50 Mt produced 1987 to 139 Mt in 2009) [16]. Such an increase in 20-years time will not be possible with present oil crops and yields and therefore more efficient and novel plant oil production systems have to be developed. The second section in this review deals with this challenge and how it can be addressed by plant lipid science.

## Competition between food and non-food in agriculture

**Figure 2:**
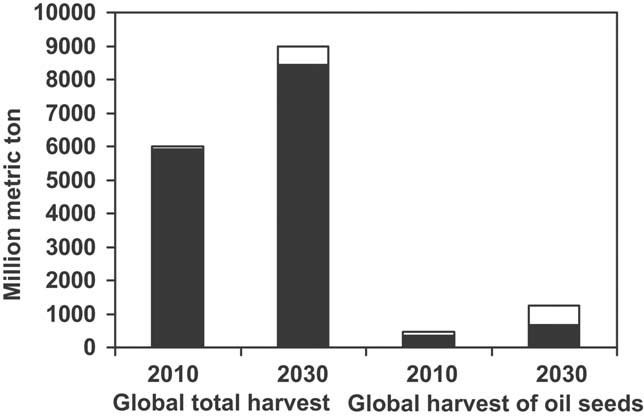
Proportion of actual and forecasted total agricultural harvest and harvest of oilseeds for chemical industry if 40% of the feedstock for chemical industry should be replaced by plant oils by 2030. Total agricultural harvest at 2030 has been forecast assuming a yearly increase of about 2%. The amount of oil seeds harvested in 2030 is derived from a forecasted global production of plant oil of 390 Mt. Values are partly based on information in the OECD/FAO Agricultural Market Outlook [140]. Open bar areas indicate oil crops harvest required for chemical industry assuming a 40% oil content of the harvest.

A valid concern about producing industrial products in agriculture is that it will compete with and could jeopardize food availability. The Food and Agriculture Organisation (FAO) estimates that food production will need to increase by 70% over the next 40 years [1]. On a 20 year timeframe we have to increase food production by about 50%, which requires about 9 Gt of harvest products. If we assume that future plant oil production systems yield an average of 40% of oil per harvested unit, a harvest of 0.57 Gt of these plants will yield 230 million oil for the chemical industry, i.e. 40% of their feedstock needs. This will correspond to around 6% of total agricultural harvest at that time (Fig. 2).

It should be noted that replacing fossil oil fuels with plant products to the same degree would require more than an order of magnitude higher proportion of total harvest. About 60% of the global harvest would equate to about 40% of the energy value of the fossil oil consumed for fuel. Assuming that the energy loss in the conversion of that harvest into fuel is about the same as making ethanol (about 50% loss), 60% of the global harvest will only substitute 20% of the fossil oil for fuel. Thus we would still have the problem of substituting the remaining 80% of fossil oil and over half of world's population would be without food.

## Optimising plant oil composition for the chemical industry

### Towards purity of a particular industrial fatty acid in seed oil

For most chemical uses, a high purity product is a prerequisite. Since plant oils are a mixture of different fatty acids, a purification step of a particular fatty acid is needed adding to both the cost and the yield of the desired fatty acid. Therefore one of the priority goals for industrial plant oils is that they should contain as close to 100% of the desired fatty acid as possible.

**Figure 3:**
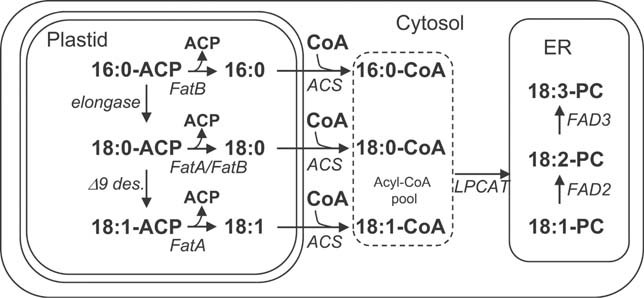
Generalised scheme of the biosynthesis of the five common fatty acids and the main enzyme steps involved. The first three fatty acids (16:0, 18:0 and 18: 1^Δ9^) are produced by de novo synthesis and desaturation in the plastids. Elongation and desaturation are carried out while the fatty acids are attached to acyl carrier protein (ACP). After removal of the ACP group by acyl-ACP thioesterases (FatA or FatB), the fatty acids are exported from the plastid and incorporated into the cytosolic acyl-CoA by the action of an acyl-CoA synthetase (ACS). 18:1^Δ9^ is then acylated onto PC, mainly by the action of the LPCAT. Further desaturations of the 18:1^Δ9^ to 18:2^Δ9,12^ and 18:3^Δ9,12,15^ are catalysed by FAD2 and FAD3 while the acyl substrates are acylated to PC. The further synthesis of glycerol lipids from acyl-CoA are depicted in Fig. 4

**Figure 4:**
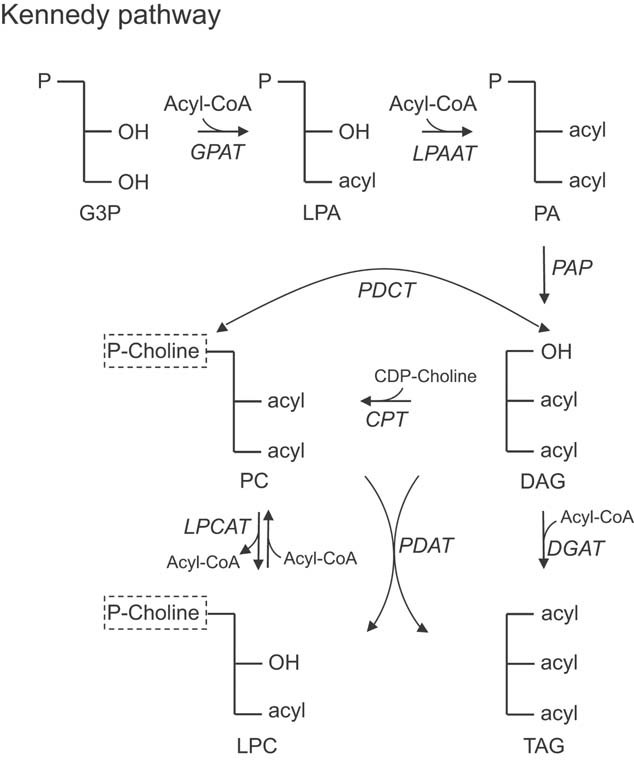
Overview of the production of storage oil in plant seeds. The acyl-CoA dependent acylation of glycerol-3-phosphate (G3P), first to lysophosphatidic acid (LPA) and then to phosphatidic acid (PA), is catalysed by the enzymes GPAT and LPAAT. Phosphatidic acid phosphatase (PAP) catalyses the dephosphorylation of PA to produce DAG and the final acylation to TAG is catalysed by DGAT. DAG can also be converted to PC by choline phosphotransferase (CPT) and/or PC/DAG cholinephosphotransferase (PDCT). TAG can also be formed by direct transfer of the acyl group from PC to DAG via the action of phospholipid/PDAT. LPCAT channels fatty acids into PC for desaturation by acylation of LPC. In the reverse reaction of LPCAT, PUFA can be transferred out to the acyl-CoA pool for utilisation in TAG synthesis.

Figure 3 depicts schematically the biosynthesis of the five common fatty acids present in the oil of our annual oil crops and the main enzyme steps involved. The proportion of 16 carbon to 18 carbon fatty acids and the proportion of saturated to unsaturated fatty acids are determined by plastidic localised enzymes (acyl–acyl carrier protein (ACP) thioesterase of FatB type and stearoyl-ACP desaturase). Conversion of oleic acid to the PUFA, linoleic and further to ALA, are catalysed by fatty acid desaturase2 (FAD2) and fatty acid desaturase3 (FAD3) enzymes and takes place on the endoplasmic reticulum (ER) in the cytosolic compartment where the substrate acyl groups are esterified to the membrane lipid phosphatidylcholine (PC). A number of enzymes are involved in the acylation of the glycerol backbone to yield TAGs, and in the shuttle of fatty acid into and out of PC (Fig. 4). It should be noted that the biosynthesis of TAGs and membrane lipids share DAG as a common precursor. This fact has special implications for the attempts to manipulate the fatty acid composition of the oil. Similar changes in fatty acid composition of membrane lipids as in TAGs are likely to occur unless counteracted by manipulation of the enzymes responsible for the flow of acyl groups into and out of the membrane lipids.

#### Manipulating the levels of the five common fatty acids

##### High oleic oil

It has been shown in a number of plant species that a drastic increase in oleic acid in the oil can be achieved at the expense of the PUFA by mutating or genetically down-regulating the oleoyl Δ12 ER desaturase (FAD2). In all of these cases, however, some PUFA remain. It is unclear if these PUFA result from residual FAD2 activity in the ER or are derived from fatty acid synthesis in the plastids/pro-plastids of the seed. There are today commercial varieties of mutated sunflower [17] and mutated or genetically modified soybean with 80–85% of oleic acid in their oils [18, 19]. In addition to these mutated or engineered oil crops, safflower (*Carthamus tinctorius*) and niger (*Guizotia abyssinica*), which usually have over 70% of linoleic and 5–10% of oleic acid in their seed oils, have rare natural alleles for high oleic traits in their gene pool, which when combined give rise to plants with 80–90% of oleic acid [20].

Further increase in oleic acid in the oil may be possible by silencing FatB and thereby lowering the amount of saturated fatty acids produced by the plastid [18, 21] as well as silencing plastidial FAD6 desaturase to lower any contribution of polyunsaturated fatty acid from the plastids. A complete absence of PUFA might however have deleterious effects in the seed, since corresponding fatty acid changes as engineered in the oil will also occur in the membrane lipids of the seed and might affect the overall membrane functions (Fig. 4). Recently, a gene for a novel enzyme was identified in Arabidopsis that might provide a way for decreasing the PUFA in the oil without decreasing it in membrane lipids [22]. This enzyme, a phospholipid/DAG cholinephosphotransferase (PDCT) transfers the phosphocholine group from PC to a DAG, thereby inter-converting the PC and DAG (Fig. 4). The DAG formed from PC can be used in TAG synthesis. Mutating this gene in Arabidopsis leads to a 40% reduction in PUFA in the seed oil concomitant with a substantial increase in the proportion of these fatty acids in PC. If PDCT plays the same important role in channelling PUFA from PC to TAG in oil crops as it does in Arabidopsis, silencing it might, in addition to lowering the levels of PUFA in the oil, restore any malfunction of the membranes caused by inadequate amounts of polyunsaturated acyl groups in the membrane lipids. Another pathway for the channelling of polyunsaturated fatty acid from PC to TAG is catalysed by the phospholipid/DAG acyl transferase (PDAT) enzyme [23, 24] and blocking this activity, preferably in combination with blocked PDCT, might further increase oleic acid in the oil (Fig. 4).

Lysophosphatidylcholine acyltransferases (LPCATs) enzymes have been postulated to be the main enzymes responsible for the channelling of oleic acid into PC for desaturation by acylating it to lysophosphatidylcholine (LPC) as well as, in the reverse reaction, transferring the PUFA out to the acyl-CoA pool for utilisation in TAG synthesis by the glycerol 3-phosphate pathway [25, 26]. There are at least four Arabidopsis enzymes that have been shown to catalyse acylation of LPC [27, 28] and their relative role in oil seeds is not yet known. In addition to their hypothesised role in exchanging acyl groups between PC and acyl-CoA, they are also important for membrane editing via the Lands cycle [29], i.e. reacylation of lysophospholipids formed by phospholipase A2 activities. Although in theory, down-regulation of LPCAT enzymes should, as for PDCT, lead to depressed levels of PUFA in the oil and higher amounts of them in the PC, it is not known if such down-regulation will affect vital membrane lipid metabolism in the seed due to impaired membrane lipid repair.

##### High PUFA oil

Oils rich in PUFA are mostly associated with healthy fish oils for dietary use. The interest for producing high amount of linoleic or ALA in oil crops has been rather limited except for serving as precursors for very long chain ω-6 and ω-3 fatty acids [30] and therefore not many such attempts are reported. However, very long chain ω-3 fatty acids found in fish oil have also been considered as a starting material for developing biodegradable polymers [31]. The high degree of unsaturation of fish oil makes it interesting as a potential monomer for polymerisation. For industrial use, linseed oil, rich in ALA (45–60%), is widely used because of its low oxidative stability, a desirable property as a drying agent in paints, varnishes and coatings [32], but which makes it less suitable for food purposes. ALA is also useful in production of polyols, which can be used in the production of foams, coatings, adhesives, sealants and elastomers. Functional groups such as hydroxyl, epoxy or carboxyl groups can be introduced at the positions of the double bonds by chemical modification, such as ozonolysis and hydrogenation. These reactive materials can then be utilised in producing valuable polymeric materials [33] – for example, soybean oil is commonly used for preparation of polyols [34].

Up-regulating the ER oleoyl and linoleoyl desaturases by over-expressing these genes in oil seeds has lead to substantial increase in PUFA in the oil. A striking example is the overexpression of a fungal bifunctional Δ12 and Δ15 desaturase in soybean, resulting in somatic embryo oils with over 70% of ALA, compared to 19% in wild type [35]. It should also be pointed out that there are natural accessions of safflower that have up to 90% of linoleic acid in their seed oil [36].

##### High palmitic and stearic oils

The amount of the saturated palmitic acid in the oil is mainly controlled by the balance of activities between two key plastidic enzymes that utilise 16:0-ACP as a substrate, namely KASII responsible for elongating 16:0-ACP to 18:0-ACP, and the FatB thioesterase responsible for removing 16:0 from 16:0-ACP and allowing its export from the plastid for incorporation in TAG. By overexpression of a palmitoyl specific FatB, an increase in 16:0 in rape seed from 6% to up to 34% was achieved [37]. In cottonseed, RNAi silencing of KASII [38], resulted in palmitic acid being raised from 27 to 77% with significant amounts being also converted to palmitoleic acid (16:1). The level of stearic acid is controlled by the activity of the stearoyl-ACP desaturase in combination with the activity of the acyl-ACP thioesterases acting on 18:0-ACP (Fig. 3). Down-regulating the stearoyl-ACP desaturase by antisense led to an increase of stearic acid in rape seed and turnip rape oil from the wild type 2% up to 40% [39]. Similarly, RNAi silencing of the *ghSAD-1* gene (encoding stearoyl-ACP desaturase) in cottonseed substantially increased stearic acid from the normal levels of 2–3% up to 40% [40]. Increase in stearic acid content up to 22% was achieved in rape by transgenic expression of an 18:0-ACP specific FatA thioesterase from mangosteen (*Garcinia mangostana*) [41]. As with the manipulation of the relative proportion of C18 monounsaturated to polyunsaturated, the manipulation of palmitic and stearic acid levels in the oil will cause similar changes in the extraplastidic membrane lipids. Due to the high melting point of these fatty acids, the membrane lipids will crystallise and cause collapse of cell metabolism if the levels are too high. The severe germination problems reported in transgenic *Brassica rapa s*eeds with elevated stearic acid content [39] might be associated with impaired membrane function due to the increased level of stearic acid in the membranes. In plants engineered to have high palmitic or stearic acid it might be possible to decrease the channelling of these fatty acids from DAG into membranes by down-regulating the PDCT. However, metabolic flux studies in developing soybean indicate that much of the saturated fatty acids are incorporated directly into PC without first going through a DAG pool [42].

In conclusion, traditional plant breeding methods and genetic engineering have shown that the percentage of any of the five the common fatty acids can be increased substantially. Oleic acid levels of over 85% can be achieved in oil crops without affecting oil yield. Further increase in oleic acid is probably possible by silencing enzyme activities responsible for the channelling of PUFA from PC, their site of synthesis, to the oil. Levels of linoleic acids can reach 90% in natural safflower varieties and ALA levels of up to 74% have been achieved in GM somatic soybean embryos. With respect to saturates, up to 77% palmitic acid has been achieved in cottonseed, but enhancement of stearic acid has been less successful for reasons mentioned above, with a maximum of about 40% being achieved in transgenic plants. This suggests some biological or agronomic limitations with respect to stearic acid accumulation. A more viable strategy to obtain high levels of stearic acid is probably through complete hydrogenation of very high oleic oils, a relatively cheap industrial process.

### Production of oils with unusual fatty acids

Although manipulation of the common fatty acids can provide expanded applications in chemical industry, the greatest potential for matching the quality of seed oil to that needed for the chemical industry is to introduce novel fatty acid structures into oil crops in as high a concentration as possible. Although this goal has its particular challenges some of these acids have the potential to reach even higher levels in the transgenic oil crops than what can be achieved with the five common fatty acids. The common five fatty acids are also present in membrane lipids in every part of the plant, including the membrane lipids of the seeds. Alterations of their levels in the oil will, more or less, be mirrored in the fatty acid composition of the seed membrane lipids, and this could negatively influence seed vigour and oil yield. Many of the unusual fatty acids are, however, only found in seed oils, where they can reach very high levels, and are excluded from the membrane lipids in the wild plants that produce them. Examples of such industrially valuable fatty acids that can reach very high levels in the seed oil include ricinoleic (18:1^Δ9,12OH^) in castor bean oil (90%), vernolic acid (18:1^Δ9,12O^) in *Bernardia pulchella* oil (90%), α-eleostearic acid (18:3^Δ9c,11t,13t^) in tung tree (80%), capric acid (10:0) in *Cuphea koeneana* (95%), crepenynic acid (18:1^Δ9,12A^) in *Crepis alpina* (80%) [11, 12]. The genes responsible for the synthesis of these and a number of other unusual fatty acids have already been cloned. However, when these genes have been initially expressed in seeds of transgenic Arabidopsis as well as oil crops, they have nearly always yielded seeds with much lower levels of the unusual fatty acid than in its host plant [9].

#### Unusual MUFA

Unsaturated fatty acids can be cleaved at their double bond sites by chemical processing to give rise to products in high demand by the chemical industry, providing that the double bond is situated at the correct position. For example, 16:1^Δ6^ and 18:1^Δ6^, which constitute about 80% of all fatty acids in the seed oils of *Thunbergia alata* and *Coriander sativum*, respectively, can be split by ozonolysis to yield adipic acid (6:0) and either capric or lauric acid. Adipic acid is one of the building blocks in 6,6-nylon, which has an annual production of about 2.5 Mt and is today made from fossil oil. A high yielding oil crop with high amount of these fatty acids in the seed oil would probably be an economically favourable alternative feedstock for the production of adipic acid. The enzymes (acyl-ACP desaturases) responsible for the production of these MUFA have been identified [43, 44]. However when these genes have been expressed in transgenic plants, the amount of these fatty acids have been very low. The bottlenecks for high accumulation in transgenic plants have been discussed in detail by Suh et al. [45] and it appears that a whole set of enzymes with particular specificities will need to be introduced. At the time these studies were done about 10 years ago, this task seemed insurmountable. However with the techniques now available to identify relevant genes and to introduce multiple genes in one construct, as in the example of Petrie et al. [46], it should be feasible to substantially increase the amount of these fatty acids in transgenic oil crops.

A more recent major breakthrough in organic chemistry is the process of olefin metathesis, using new types of catalysts. Its use on unsaturated fatty acids has made it possible to easily achieve a range of useful chemical products which were not possible with more classical methods [47, 48]. For example, ω-7 unsaturated fatty acids such as palmitoleic (16:1^Δ9^) and *cis*-vaccenic acid (18:1^Δ11^) could be split to yield an ω-unsaturated fatty acid and 1-octaene, which has great value as a chemical feedstock. Very recently, a major breakthrough in the production of ω-7 fatty acids in transgenic plants was reported [49]. The work is of particular interest since the authors introduced a 16:0-ACP desaturase that was engineered to convert 16:0 to 16:1^Δ9^ with a more than 100-fold higher specificity than that of naturally occurring enzymes. Expression of this gene together with a cytosolic 16:0^Δ9^ desaturase in Arabidopsis plants carrying a mutation in the KASII condensing enzyme (responsible for the elongation of 16:0–18:0) increased the levels of ω-7 fatty acids in the seed oil up to 71%. This is about the same level as in *Doxantha unguis-cati*, the plant with the highest recorded amount of ω-7 fatty acids in its seed oil [49].

#### Production of medium chain fatty acids

Fatty acids shorter than C16 have substantial industrial uses. Lauric acid, the dominating fatty acid in palm kernel oil and coconut oil is, as mentioned earlier, an important feedstock for the detergent industry. However, shorter chain fatty acids like caprylic acid (8:0) and capric acid currently have no commercially viable plant source. These fatty acids are fractionated out from palm kernel and coconut fats and used mainly in the cosmetic industry as caproyl/capric TAGs. For the chemical industry, the same fatty acids are used in organic synthesis of a multitude of compounds and are mostly made from fossil sources via oxidation of the corresponding aldehyde. An oil crop producing high amounts of caprylic or capric acid in its oil would be an economic viable alternative to the organic chemical synthesis.

**Figure 5:**
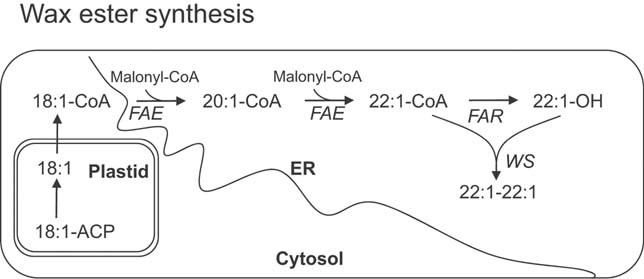
Schematic diagram of the biosynthesis of 22:1–22:1 wax esters. The membrane bound FAE elongates the 18:1-CoA in two steps on the cytosolic side of the ER membrane, after which the 22:1 fatty acid is reduced to 22:1 alcohol in two steps catalysed by a FAR. In the final step, a WS esterifies a fatty acid to a fatty alcohol to produce the 22:1–22:1 wax ester.

Medium chain fatty acids (8:0–14:0) are produced in some plant seeds by special acyl-ACP thioesterases that cleave the fatty acid from its ACP moiety at C8, C10, C12 or C14 stages during de novo fatty acid synthesis [50–52]. Once the free fatty acid is formed, it is exported out from the plastid and acylated to glycerol through the glycerol 3-phosphate pathway in the cytosol (see Fig. 3 and 5) to yield medium chain TAGs. One of the earliest and most remarkable engineering of oil quality was the production of over 60% of lauric acid in rape seed [53]. This was done by transgenically expressing a single gene encoding a 12:0-ACP specific thioesterase obtained from Californian bay tree. Commercial production of GM lauric acid producing rape occurred for a few years in USA, but ceased because it could not economically compete with lauric acid production from the palm sources. Introduction of thioesterase genes with specificities towards C8 and C10 acyl-ACPs from different *Cuphea* species into transgenic oil crops yielded much less of the medium chain fatty acids, with caprylic acid levels of up to 8% and capric acid levels of 30% [53]. Biochemical studies of the properties of enzymes involved in the acylation of the medium chain fatty acids to the glycerol backbone from *Cuphea* species and species not accumulating medium chain fatty acids [54–56], analysis of the acyl-CoA pool in *Cuphea* seeds and transgenic seeds producing medium chain fatty acids [57], and the observed increase in β-oxidation in these transgenic seeds [58, 59], all suggest the same bottlenecks in achieving higher amount of medium chain fatty acids in transgenic seeds. The transgenic seeds lack enzymes that efficiently acylate the medium chain fatty acids onto TAG backbones, which leads to a build up of these fatty acids in the acyl-CoA pool and their resultant shunting to β-oxidation. Of particular importance in the wild plants appears to be the concerted action of specialised glycerol-3-phosphate acyltransferase (GPAT) and lysophosphatidic acid acyltransferase (LPAAT) that allows the production of exclusively di-medium chain DAGs, which are preferentially used in the last acylation step by the DGAT to form TAG [54, 55]. It has further been shown that plant cells are capable to import medium chain fatty acids into the plastids and further elongate them and that this elongation can be reduced by lowering the activity of the acyl-ACP synthase [60]. It could therefore be anticipated that much higher levels of medium chain fatty acids in transgenic seeds could be achieved by co-expressing the thioesterase with the *Cuphea* GPAT, LPAAT and DGAT enzymes in combination with down regulation of the endogenous acyl-ACP synthase activity.

#### Very high amount of erucic acid

Erucic acid is used to produce erucamide, which is used as a slipping agent for production of extruded polyethylene and propylene films such as shopping or refuse bags [61, 62]. Erucic acid is now mainly derived from high erucic rape, which has 45–55% of erucic acid in its oil. Erucic acid is produced by elongation of oleic acid in the form of acyl-CoA in the cytosol (Fig. 5) and is a TAG specific fatty acid and thus is not present in the membrane lipids of the seed. In Brassicaceae oil crops, the levels of fatty acid longer than 20 carbons never exceed 66% with the highest amount found in *Crambe abyssinica*. It has been shown that erucic acid is only found in the outer positions of the TAGs in these species and biochemical investigation of the properties of LPAAT, the enzyme responsible for the insertion of fatty acids in the *sn*-2 position of the glycerol backbone, from rapeseed showed that this enzyme could not use erucoyl-CoA as the acyl donor [63]. *Limnanthes douglasii* is a plant that has over 95% of very long chain fatty acids (≥20 carbons) in its seed oil. This oil is mainly composed of 20:1^Δ5^ fatty acids but also some erucic acid, which is also found in the *sn*-2 position of the TAGs. It was shown that the *Limnanthes* LPAAT readily utilised erucoyl-CoA [64] and expression of its encoding gene in rape seed led to redistribution of erucic acid from the outer *sn*-1 and *sn*-3 positions to the middle *sn*-2 position [65]. However, the overall level of erucic acid did not increase, even when combined with overexpression of the rapeseed fatty acid elongase gene (FAE), responsible for the elongation of oleic acid to erucic acid. In contrast, when transgenic rape over-expressing the FAE and *Limnanthes* LPAAT was crossed with a rapeseed line with greatly increased 18:1 levels (resulting from mutations), levels of erucic acid up to 72% were obtained in the offspring and the total amount of very long chain fatty acids accounted for up to 77% of all fatty acids [66]. Further increase of erucic acid could possibly be achieved by blocking pathways channelling remaining oleate, via acyl-CoA independent pathways into the oil as discussed for the high oleate trait above. However, the availability of the cytosolic pool of malonyl-CoA needed for fatty acid elongation and other enzymes than the condensing enzyme (FAE) in the elongation complex also need to be considered as possible limiting factors.

#### Unusual fatty acids produced on phosphatidylcholine

For enzymes producing unusual fatty acids from precursor fatty acids esterified to PC, the major bottleneck seems to be the channelling of the unusual fatty acids from PC to the TAG [9]. These fatty acids include the hydroxylated fatty acid (ricinoleic acid), epoxy fatty acids (vernolic acid), conjugated fatty acids (α-eleostearic acid) and acetylenic fatty acids [67, 68], as well as cyclopropene fatty acids [69, 70].

Significant increase in the production of ricinoleic, vernolic and α-eleostearic acid has been achieved by combining the expression of the enzyme for the production of the fatty acid with expression of a DAG acyltransferase (DGAT) from a species naturally producing these acids [71–73]. It can be anticipated, based on biochemical studies [56], that the DGAT will preferentially acylate DAGs with the unusual fatty acid, possibly in combination with acylating them preferentially with unusual fatty acids derived from acyl-CoA, and thereby increasing the flow of these acids out from PC and releasing the product inhibitory effect these fatty acids seem to exert on the enzymes producing them [68]. Despite this, the levels of these fatty acids in the oil of unusual fatty acids in the transgenic plants have so far not exceeded 30%, which has to be compared to 80–90% in the host plant.

Candidate enzymes for further increasing the levels of these unusual fatty acids in transgenic plants are PDCT, PDAT, LPCAT, LPAAT, specific phospholipases and GPAT [9, 74, 75]. Since genes are identified for the first four types of enzymes, their effect in transgenic plants are now being evaluated. A recent result of this work is the co-expression of castor bean *RcPDAT1A* and *RcDGAT2* with the hydroxylase *RcFAH12* in arabidopsis. Combining *RcPDAT1A* and *RcFAH12* resulted in an increase of ricinoleic acid in the seed oil from 17 to 27% [76], but a reduction in total oil content. Additional expression of *RcDGAT2* restored the oil content to nearly wild type level and gave rise to major increase in the mass of hydroxy fatty acids accumulating in the seed.

A possible further bottleneck is the amount of the fatty acid modifying enzyme in the transgenic plants. It has been shown in plants that naturally accumulate unusual fatty acids, that mRNA levels for these enzymes are expressed at similar levels to storage proteins [72, 77] and it is uncertain if such levels are obtained in the transgenic plants. Another aspect that has to be taken into account is that these enzymes need cytochrome b5 and b5 reductase for their activity. It has been shown that down-regulating cytochrome b5 reductase lowers the hydroxylation of oleic acid to ricinoleic acid in Arabidopsis seeds but has little effect on the desaturation of oleic acid to linoleic, a reaction which also requires this reductase [78]. Cytochrome b5 has also been shown to be involved in 2-hydroxylation of long chained sphingolipids [79] It could thus turn out that many more genes might have to be introduced, and perhaps also endogenous genes silenced, before transgenic plants with around 90% of these unusual fatty acids in the oil will be obtained. However, there is no reason to believe that it cannot be achieved, given sufficient research and time.

#### Unusual TAG structures

Due to the high viscosity of TAGs, they cannot be used directly as biodiesel – instead the fatty acids have to be converted into their methyl esters. Burning Bush (*Euonymus alatus*) accumulates embryo and endosperm oils which only have two fatty acids esterified to the glycerol, with the third hydroxy group occupied by an acetate group. The kinematic viscosity of this oil is decreased by some 40% compared to TAGs having only long chain acyl groups and reaches a value similar to Diesel Grade no. 4-D [80]. Thus, such an oil could probably be used directly in some diesel engines. By expressing a DGAT gene from Burning Bush in Arabidopsis, seed oils with up to 40 mol% of acetyl containing TAGs was achieved [80]. When the same gene was expressed in an Arabidopsis mutated in a DGAT gene, seeds with over 60% of these TAG molecules were obtained (Timothy Durrett, MSU, personal communication). Although we do not favour the long-term use of plant oils for fuel, the work, together with the ω-7 and lauric fatty acids mentioned above, represent the best three examples so far of how similar high levels of unusual and valuable chemical structures from naturally occurring plant oils can be achieved through biotechnology in transgenic plants.

### Non-TAG oils in transgenic crops

TAGs have many attractive features as lubricants but have limited ability to replace fossil oil based lubricants due to poor hydrolytic and oxidative stability. In contrast wax esters, which are esters between a fatty acid and a fatty alcohol, have excellent resistance to hydrolysis [81]. Spermaceti oil (from the spermaceti whale) is mainly composed of wax esters and was widely used in high pressure and temperature lubricants before a global ban on hunting the whale was introduced in 1972. An alternative biological source of wax esters could be the desert shrub jojoba (*S. chinensis*) which uniquely has seed oil consisting almost entirely of wax esters. However, the jojoba plant is low yielding and requires high labour input for harvest and therefore the wax esters extracted from this plant are very expensive and unable to compete with cheaper petroleum based products in the lubricant market. Today they are only used in cosmetics and other very high value products. In principle, only two additional enzymatic activities need to be introduced in order to produce wax esters in a seed cell – a fatty acid reductase (FAR) that in a two step reduction process converts a fatty acid to a fatty alcohol, and a wax synthase (WS) that esterifies the fatty alcohol to a fatty acid (Fig. 5).

By expressing the jojoba FAR and jojoba WS in combination with a FAE in Arabidopsis, the Calgene company reported that a major proportion of the TAGs in the seeds were replaced by wax esters [82]. The jojoba type of wax esters are mainly composed of monounsaturated 20 and 22 carbon chains and have a melting point (about 9°C) that is too high to be suitable for lubricant use in cold climate. However wax esters of very diverse composition are found in all phyla and thus a vast pool of FAR and WS genes are available to choose among to produce different kind of wax esters, suited for lubrication under various conditions and other applications. In addition, wax esters can also be hydrolysed to yield fatty acids and fatty alcohols, of which the latter have substantial use in chemical industry and an economic value about twice that of the fatty acid. However, in order to achieve efficient accumulation of wax esters in a seed using these genes, a number of aspects have to be considered. For example, the fatty acids available in the seeds should have the optimal profile for conversion to the desired wax ester, and the FAR and WS must have the right specificity and sub-cellular location for efficient synthesis. Additionally the seeds need to be able to mobilise the novel wax esters during germination. The world-wide biotechnology project ICON (Industrial Crops producing added value Oils for Novel chemicals; http://icon.slu.se/) is now addressing these challenges in order to produce different types of wax esters in dedicated industrial oil crops. Since wax ester biosynthesis, in contrast to TAG biosynthesis does not share any glycerolipid intermediate with membrane lipid synthesis, it might be a more viable way to accumulate high amounts of those acyl groups that, if present in membranes, would severely disturb cellular functions. It should be noted that more than 90% of the carbon chains in jojoba oil are 20 carbon or longer.

In addition to TAGs and wax esters, there is a vast array of industrially interesting extracellular lipids occurring in plant tissues such as in cutin, suberin and the wax layer, e.g. ω-hydroxylated fatty acids, dicarboxylic acid, fatty aldehydes, alkenes and alkanes. Recently much has been learnt about the accumulation of these compounds and the genes and enzymes that direct their biosynthesis [13, 14]. It is also conceivable that synthetic biology could be used to develop and accumulate completely novel lipids in plants, ones that are not yet occurring in nature. This could be done by combining different lipid metabolising enzymes in novel ways, analogous to the successful application of synthetic biology in microorganisms to produce chemical feedstocks such as propanediol [83]. The production of any such compounds in transgenic plants however poses specific problems. Over-producing and targeting them extracellularly might severely affect the agronomic performance of the plant, such as resistance to biotic and abiotic stresses. Producing them inside the cell, such as in a seed cell, requires that the product does not harm other cell functions and should be able to be utilised as energy when the seeds germinate. Some of the novel plant oil production systems, such as induced biosynthesis in leaves, as discussed in the next section, might be more suitable for the accumulation of such exotic lipids.

## Increasing plant oil production

Although optimising the composition of the plant oils can make them more competitive in the chemical industry there is, as mentioned above, an additional need to triple the present plant oil production in 20 years time to be able to replace 40% of the fossil oil in the chemical industry without compromising the supply of plant oils for the food sector. The total acreage of cultivated land has only marginally increased in the last 30 years and most of the increase in agricultural production has largely been due to the introduction of more efficient production methods and higher yielding crops, i.e. what has been called the Green Revolution. Major acreage increase cannot be reasonably expected in the next 20 years, since expansion of arable land would require inroads into forest areas, reducing the availability of an important biobased feedstock [84], and increasing greenhouse gas emissions [85]. Thus, we must increase the overall agriculture yield per hectare by about 50% over the coming 20 years and at the same time triple the oil component. Although traditional plant breeding in combination with improved agricultural practices has steadily increased oil crop yields, this increase appears to have plateauxed and thus new ways to increase oil yield will have to be introduced in order to reach the goal of tripling plant oil production [86].

The opportunities for increasing oil yield through biotechnology are classified below into three strategies: (i) increasing oil content in major oil crops; (ii) redirecting sucrose or starch synthesis to favour oil accumulation; and (iii) oil accumulation in vegetative tissues. We outline how science is addressing these strategies, what has been achieved so far and what can be expected in the future. It should be noted that new biotechnological strategies for improving plant resistance to abiotic and biotic stresses and improved agronomic practices will also be important in increasing productivity through reducing crop losses.

### Increasing oil content in major oil crops

Three oil crops make up 77% of the plant oil production today, oil-palm, soybean and rapeseed [87]. The oil content per dry weight in the mesocarp of oil palm is around 80% and thus there is little prospect for further increase since a minimum proportion of structural cellular components are needed to enclose this oil. Such high oil content can probably not be achieved in seeds since they need more cell components for prolonged desiccation, such as protective seed coat and cell walls that can withstand mechanical stress, as well as they have to have nitrogen reserves (storage proteins) for germination. However many oilseeds have oil contents that fall well short of the maximum seed oil contents, and there is thus significant opportunity to raise oil content in several oilseeds. Using biotechnology, different approaches to increase the oil content in seeds have been studied including up-regulating fatty acid synthesis, and modifying expression of individual TAG biosynthetic enzymes and transcription factors. The reported gain in accumulated oil varies depending on the type of enzyme studied or approach used, but few have reached more than a 50% increase in oil content, as was shown in a recent comprehensive review by Weselake et al. [88]. Many of the approaches used have focused on manipulating enzymes involved in the pathways of fatty acid synthesis. An interesting exception is the work by Vigeolas et al. [89] that achieved a 40% increase in the fatty acid content of the seeds by over-expressing a class 2 haemoglobin (AHb2), which is not integral to these pathways. This effect was explained as a consequence of the increased energy state in the seeds resulting from the increased AHb2 expression.

Larger seeds can have higher oil content than small seeds because of their larger volume to seed coat surface ratio. Among the highest oil content recorded for larger seeds are Brazil nut and Macadamia nut with about 70% of oil [90] and for smaller seeds such as sesame with up to 60% [91]. The latest rape seed cultivars have approximately 50% of oil [92] and it is not likely that this could be increased to more than 60%. However, soybean has only 20% oil and it should theoretically be possible to triple that content to 60%. The major component of soybean is the economically valuable protein accounting for approximately 40% with about 30% of carbohydrates and 10% of fibre. A small (1.5%) increase in oil content in soybean has already been achieved without compromising the yield of protein by expression of a fungal DGAT2 [93]. Since many of the carbohydrates are important for preventing seed deterioration during desiccation [94], substantially increased oil content will need to be mainly at the expense of the protein content. While this is currently neither an economically viable option or desired for global food security reasons, this situation could change in the future as a result of potentially increasing plant oil prices as crude oil supply declines, and increased availability of alternative feed protein sources as by-products of biofuel production. Another potential target for increasing oil content is the lupine. Lupine seeds (from a number of species) are today only used in feed and have a much lower market value than soybean. There are lupine varieties that have similar content of protein and oil to soybean and seed yields can be nearly twice that of soybean [95]. A major portion of the amino acids used in seed storage protein synthesis are imported via the phloem, whereas the oil is synthesised from imported sucrose [96]. Thus in order to redirect the use of the photosynthate from amino acid synthesis into oil requires not only an alteration of the metabolism in the seeds but also the metabolism in the photosynthetic leaf and pod tissues, as well as in the loading mechanism to and from the phloem. Recently Zhang et al. [97] have shown that an amino acid permease 2 (AAP2) enzyme is located to the phloem and is highly important for phloem loading and amino acid distribution to the embryo. Furthermore, *AAP2* T-DNA insertional mutant lines with an altered N partitioning within the plant showed increased carbon assimilation and export from the leaf for sink carbon supply, and also had elevated seed fatty acid levels and strong increases in the number of branches and siliques as well as in seed yield. It is well known that increased amount of nitrogen fertilizer reduce the oil/protein ratio of the seed but generally increases the total oil yield/ha due to increased seed yield [98–100]. The results by Zhang et al. [97] are therefore promising and support the general idea of increased oil yield through modification in the nitrogen supply to the developing seed and highlight the importance of AAP2 for this mechanism.

### Redirecting sucrose or starch synthesis to favour oil accumulation

As previously stated, agriculture is relying on three plant species for a greater part of the plant oil production. Oil palm acreage increase is limited due to the need for preservation of biological diversity and likewise soybean and rapeseed acreage is ultimately limited by the need for crop rotation in order to manage pest and pathogen problems. It would be highly desirable to develop additional new high yielding oil crops to support sustainable expansion and diversification of oil production.

All plants accumulate oil in some of their tissues. Although not oil crops by definition, both potato tubers and sugar beet seeds contain oil, and cereals have about 25–35% of oil in their embryo tissue, which usually accounts for only a small percentage of the seed weight. Thus, all plants have genes for oil biosynthetic pathways and also transcription factors for activating the genes in a coordinated fashion for oil synthesis. If these transcription factors could be activated in sink tissues it might lead to conversion of the loaded sucrose into oil instead of being accumulated in the vacuoles, as occurs in sugar beet, or converted to starch, such as in starch producing tubers and grains. Thereby sugar and starch accumulating crops could become very productive oil crops.

Maize production is more than 800 Mt per year [101] and although it is not regarded as an oil crop, it has higher seed oil content than most other cereals, on average 4%, corresponding to a maize oil production of 32 Mt annually. Although only a minor part (7%) of this oil is currently extracted, 32 Mt of oil is actually nearly double the amount of rape seed oil produced in the world and could replace nearly 10% of the fossil oil used in the chemical industry today. The oil in the maize kernel is nearly totally confined to the embryo and scutellum where it constitutes about 35% of the weight. It has recently been shown that this percentage can be increased to 45% by over-expressing the ZmWRI1 gene, a maize transcription factor [102] homologous to the Wrinkled1 transcription factor that has been shown to regulate many of the glycolytic and fatty acid biosynthesis genes in Arabidopsis seeds [103]. Most importantly, when this maize gene was over-expressed in elite maize hybrid cultivars and field tested at various sites, they gave 22% more oil yield without any decrease in overall grain yield. When the transgenic embryo was analysed for its components it was shown that starch content was reduced (from about 8 to 2.5%) but this reduction accounted for only one third of the sugars needed for the increased oil content. It thus appeared that overexpression of the *ZmWRI1* increased the total sugar allocation into the embryo. It is obvious from many studies that the potential production of photosynthate is not usually the limiting factor for the accumulation of storage product in the sink tissue, but the strength of the sink is important. A striking example of that is that the total sugar loading in sugar cane stems can be doubled by expressing a gene for the isomerise that converts sucrose into isomaltulose, leading to unchanged levels of sucrose and production of an equivalent amount of isomaltulose [104]. This indicates that the sink cells in sugar cane do not recognise isomaltulose as a storage compound, and thereby signal for increased photosynthesis, sugar transport and sugar loading.

The main storage product in maize kernel is starch, making up about 65% of the weight, of which nearly all is confined to the endosperm. Both starch and oil use sucrose as starting material and expressing the *ZmWRI1* gene in the embryo led to oil accumulation partly at the expense of starch. Attempts were therefore made to further increase oil content in maize by over-expressing the *ZmWRI1* in the endosperm, but this did not result in any accumulation of oil in this tissue [102], for reasons that were not further investigated. It should however be noted that another cereal, oat, has even higher oil content than maize and the majority of this oil is in the endosperm [105]. The oil content of the grain of certain lines of oat can reach as high as 18% [106] and is negatively correlated with starch content [105, 107]. If similar oil content could be reached in maize kernels, the potential global annual production of maize oil alone could exceed the total global vegetable oil production of today, assuming that no penalty in yield was seen.

**Figure 6:**
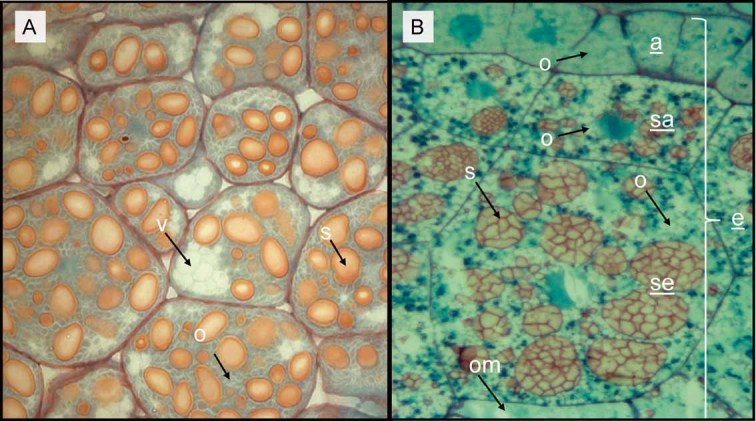
Light microscopy pictures of (A) nutsedge tubers at 35 days after tuber initiation and (B) endosperm tissue from developing oat grain. In (A) oil is seen as discrete grey bodies (o), starch grains (s) are visible as white or reddish structures enclosed by amyloplast, and remaining vacuoles (v) as areas with white vesicles. In (B) the different sub-layers of the endosperm (e) are marked as aleurone (a), sub-aleurone (sa) and the starchy endosperm (se). Starch granules (s) are present in sub-aleurone layer and starchy endosperm but not in the aleurone layer. Oil is present as oil bodies (o) in the aleurone and sub-aleurone layer, either as oil bodies (o) or confluent oil masses (om) in starchy endosperm.

Endosperms in dicotyledonous plants are living tissue. Those plants that accumulate endosperm oil metabolise this oil through β-oxidation into sugars in the endosperm cells during germination and shunt these sugars into the growing embryo. Endosperm cells in monocotyledonous plants, such as maize on the other hand, undergo programmed cell death during seed maturation and thus cannot develop glyoxysomes for β-oxidation during germination. This raises the question of whether the oil in the cereal endosperm is a waste product, making high endosperm oil cereals energy deficient during germination. However, it was shown that in germinating oat seeds (Fig. 6), the endosperm oil was hydrolysed to free fatty acids that were effectively absorbed by the scutellum and partly further shunted into the embryo where they were beta-oxidised to provide the embryo with energy [108]. This mobilisation resembles the way in which oil in the endosperm in monocotyledonous oil rich palm seeds is mobilised [109]. Conceptually this demonstrates the feasibility of converting oats, and perhaps also other cereals, into true oil crops, once the regulatory genetic switches governing this synthesis are discovered.

Nutsedge (*Cyperus esculentus*) provides another interesting example of oil accumulation in a sink tissue that in most other plant species is devoid of this storage product. Nutsedge has about 25% oil and 30% starch (of dry weight) in its stem tubers (Fig. 6), a tissue that usually is devoid of oil in other plants, such as the potato tubers. It has been shown that starch and oil are accumulated simultaneously in the same nutsedge tuber cells during tuber filling [110]. Identifying the transcription factors that regulate the orchestration of oil biosynthesis in these tubers might give tools for redirecting the sucrose to oil in many tuber or tap root crops. For example, sugar beet yields about 7 t of sugar/ha, which converted to oil would give about 3 t of oil/ha (oil being 2.2 times more energy dense than sugar) which is about double yield of oil from winter rapeseed.

The availability of oat and nutsedge as model systems for oil biosynthesis in endosperm and tubers, respectively, combined with the rapid and relatively cheap sequencing of whole transcriptomes available today, opens up the possibility to identify candidate genes for the genetic switches in sugar utilisation for the different storage products in sink tissues.

### Oil accumulation in vegetative tissues

Plants have evolved the capacity to accumulate large amounts of storage products in tubers and seeds in order to ensure the availability of sufficient energy for propagation and the establishment of new generations. Through plant breeding, the levels of these storage products in sink tissues have been enhanced far beyond what is needed for the survival of the species. However, the sink organs developed for plant survival might not be the optimal choice for maximal accumulation of a storage product per area of cultivated land. Plants use much energy and time in building the infrastructure, i.e. leaves, stem and roots, to support the sink tissue with sugars during a limited life time of the plant. A good measure on how much of the accumulated energy or carbon is contained in the sink tissue of the plant is given by the harvest index of the crop [111]. Harvest indices vary between 0.1 and 0.6 depending on the crop and show an even greater variation in single crops due to different abiotic and biotic factors [112]. During day-time, leaves produce an excess of sugars that are stored as starch and which are utilised during the dark hours in respiration and growth. The amount of starch produced during a day can reach more than 10% of the dry weight of leaf [113]. If starch mobilisation during the dark hours were inhibited to 50%, this would severely affect plant development; but if induced when leaves are fully developed and before sink tissue had started to develop, the leaves should, theoretically be able to accumulate 50% of their dry weight as starch within 10 days. Since the dry weight of leaves in broad leaf crops, such as tobacco, can reach 20 t/ha [114], it could thus be theoretically possible to achieve a yield of leaf starch of 10 t/ha. If instead this stored carbon was channelled into oil, this would translate to about 4.5 t of oil/ha, which is approximately equal to the oil yield of oil palm.

Although photosynthetic tissues are not recognised as oil producing, leaf mesophyll cells of many species have been found to contain oil droplets in the cytosol [115]. TAG content follows a diurnal pattern which suggests a metabolic function of temporary energy storage similar to what can be found for the major temporary storage compound starch [116]. TAG levels up to 5 mg/g fresh weight were found in leaves of *Lactuca serriola*, which would translate into about 5% of dry weight [116]. TAG can also be found in leaf tissue at senescence [117]. Additionally, some TAG is produced in leaf tissue at the onset of senescence with chloroplast structure degradation and proliferation of plastoglobuli. Presumably this is as a way of taking care of carbon already fixed in membrane lipids or perhaps specifically thylakoid galactolipids. The TAG produced during senescence is not accumulated but there is a rapid turnover of fatty acids and other components during this phase. One strategy to bring about accumulation of TAG in vegetative tissues could be to reduce TAG turnover by blocking β-oxidation. Mutants for *COMATOSE* (*CTS*), coding for a peroxisomal ABC transporter in Arabidopsis, were shown to accumulate TAG up to 2% of dry weight as compared to very marginal amounts (below 0.1%) in wild type [118]. Another interesting strategy involving disruption of normal lipid remodelling and turnover to cause a greater maintenance of cytosolic leaf TAG has its origins in the mammalian lipid storage disorder, associated with the *CGI-58* gene, where cells accumulate lipid droplets which they normally do not do [119]. The gene product of the most similar gene in Arabidopsis, At4g24160, has been shown to be soluble and have LPAAT, TAG lipase and phospholipase activity [120]. A mutation in this locus causes a disorder in *Arabidopsis* similar to the one in humans, where lipid droplets with leaf type fatty acids are accumulated in the cytosol of vegetative tissue [121].

Cytosolic oil droplets as well as the TAG found during senescence are mainly derived from membrane lipids and thus their fatty acid profile will be the similar to that of the membranes. This fatty acid composition may not be optimal for the properties of oil to be extracted. Various strategies for increasing the oil yield in leaf tissues including adding embryonic features to the leaf TAG fatty acid profile have recently been published. One target could be to stimulate the production of typical seed fatty acids in the leaf tissue via transcription factors that turn on gene sets typical of embryo development and seed oil accumulation while at the same time having the benefit of a stimulating effect on overall accumulation of TAG. Loci named after mutations affecting seed development and maturation such as *LEAFY COTYLEDON1* and *2* (*LEC1* and *LEC2*), *FUSCA3* (*FUS3*), *ABSISIC ACID INSENSITIVE3* (*ABI3*) and *WRINKLED1* (*WRI1*), where *WRI1* is a direct target of *LEC2*, have been shown to code for transcription factors [103, 122–126]. Several of these transcription factors have been ectopically or heterologously expressed in leaves and resulted in oil production in vegetative tissues. Induced expression of *LEC2* in developed *Arabidopsis* plants resulted in a leaf lipids with a seed-like fatty acid profile as well as increased proportions of TAG [127]. *LEC1* which is reported to be partly independent of and does not regulate expression of LEC2 has also been expressed by induction in developed *Arabidopsis* plants [128]. Although some growth retardation was observed, *LEC1* was found to induce the expression of a large set of genes related to embryo or seed maturation and increase an array of fatty acids as well as promote formation of oil bodies in vegetative tissues. *WRI1* most likely operates downstream of *LEC1* and *LEC2* and induces expression of genes encoding enzymes involved in glycolysis and fatty acid biosynthesis [103, 129]. *WRI1* seemingly incurs less pleiotropic effects as compared to master regulators as *LEC1* and *LEC2*. However, on the other hand, *WRI1* seems to be dependent on high sugar concentrations to actually show an effect upon ectopic expression in Arabidopsis seedlings [122].

Even though ectopically expressed transcription factors induce some expression of oil body proteins [123, 127] it could be worthwhile considering leaf-specific expression of oil body proteins which could reduce turnover and stabilise TAG to enhance prospects of higher oil contents. The expression in yeast of an *Arabidopsis* oil body protein was reported to yield increased accumulation of oil bodies [130].

An example of initial steps towards increasing lipids in green tissues of a crop plant is the overexpression of DGAT1 and separately the induction of *LEC2* leaf expression, an embryo specific transcription factor, in tobacco [114]. The highest oil content achieved so far was in leaf with 6.8% of extracted fatty acid per dry weight, translating to 1.4 t oil/ha in fields with 20 t/ha of leaf dry weight. Since this line of attempts has just started, a combination of the different approaches can be expected to further dramatically increase the percentage oil that can be produced in leaves. In view of the impact of oil production in leaf tissue on diverting energy away from growth, and additionally the overall morphogenesis disruptions associated with ectopic expression of transcription factors such as *LEC1* [123] and *LEC2* [126], it will be important to consider utilising inducible or developmentally regulated expression of added genes to avoid disturbance of growth and development.

Much focus of this research has been on the manipulation of lipids in leaves, however other parts of the plant might also be considered for the accumulation of oil. In response to seasonal changes such as increased activities in the cambium zone during spring and decreased activity during late summer while preparing for winter dormancy, oil droplets are found in the cambium of *Populus,Salix* and other type of tree species [131–133]. A striking example of a similar phenomenon is the Mongolian firewood (*Tetraena mongolica*), that stores up to 10% oil (DW) in the phloem, which translates into around 5% in the stem [134]. A platform to explore this type of oil accumulation could be Salix that is closely related to the completely sequenced poplar and for which efficient transformation methods exist [135–137]. If the content of the already present oil in the cambium of Salix could be increased to similar levels as in Mongolian firewood it would significantly increase the economy of Salix cultivation as a bioenergy source due to the higher energy density of the plant. Sugar cane is another example of a current high yielding crop with stem storage capacity. Sugar cane transformation protocols are available and expression of transcription factors could be tested.

Since TAGs are continuously synthesised and turned over in leaves and share common pathways with membrane lipids, where content and composition need to be tightly controlled for proper metabolism, it might be a better strategy to accumulate other types of lipids in leaf systems, such as those that are found in extracellular parts of the plant and which are much more resistant to break down [138]. Furthermore, since the chloroplast is the site of fatty acid synthesis and shares many features of this synthesis in common with bacteria, reflecting its evolutionary prokaryotic history, it is of special interest to try to employ similar strategies to enhance fatty acid synthesis by diverting the acyl groups from endogenous lipid synthesis in these organelles as has already been shown to increase fatty acid synthesis by several orders of magnitude in prokaryotes [139].

## Conclusion

Plant oils have the potential to provide functionally equivalent industrial chemical feedstocks as renewable replacements for the finite fossil-based raw materials currently used, provided their composition can be engineered to meet end-use requirements, and that they can be produced on sufficient scale to meet current and growing industrial demands. Realisation of this potential is heavily reliant on three key aspects of plant lipid metabolic engineering: firstly, the tailoring of plant oils to have high purity of single desirable fatty acids (preferably >90%); secondly, the introduction of unusual fatty acids that have specialty end-use functionalities not normally present in our major oil crop species; and thirdly, significant increases in plant oil production capacity through increased oil content in current oil crops, and conversion of other high biomass crops into accumulators of oil.

Since the advent of plant molecular biology much progress has been made in unravelling the enzymatic pathways of fatty acid and oil synthesis in plants, and in elucidating the genes encoding the key enzymes in several oil plant species. Early successes in modifying the relative proportions of the main common fatty acids have now been surpassed by the engineering of production of unusual fatty acids, including examples of the introduction of complex multistep biosynthetic pathways. These advances have so far resulted in relatively small concentrations of unusual fatty acids, which although suitable for some niche food and industrial applications, fall short of the high purity required for cost-effective industrial utilisation on a larger scale. With appreciation of the need to exclude unusual fatty acids from cellular membranes and an increasing knowledge of how wild plants achieve this through enzyme specialisation, we are now entering a period where metabolic engineering research can be anticipated to provide the solutions to assembly of high purity unusual fatty acids in oil crops. Additionally, current advances in engineering synthesis of wax esters as alternatives to triglycerides in seed storage lipids show promise of providing a simplified approach to accumulation of unusual fatty acids, in this case concomitantly with production of industrially useful fatty alcohols.

Research to provide step changes in oil content in seed and other lipid accumulating tissue is clearly at an earlier stage, and is reliant on a combination of biochemical and genetic approaches. High-throughput comparative sequencing of transcriptomes from high and low oil-accumulating tissue is a promising approach to unravelling the constellations of genes, and their controlling transcription factors, associated with lipid synthesis and accumulation. With access to these genes, and an improved biochemical understanding of the pathways involved, the prospects are good for defining the metabolic manipulations needed to increase storage lipid synthesis, perhaps involving coordinated upregulation of both the fatty acid synthesis (source) and triglyceride or wax ester assembly (sink) components of the pathways. Significant enhancements in oil content in tissues that already synthesise some oils should ultimately pave the way for defining how to redirect carbon flux from other storage compounds, such as starch and protein, into storage lipids in tuber and cereal endosperm tissues and vegetative parts of high biomass plants, thus creating novel high-oil crops. A further challenge is to bring together progress in transgenic synthesis of unusual fatty acids with that in increasing storage lipid content, to ensure that very high levels of unusual fatty acids can be achieved. Thus compatible technological approaches need to be found in both dimensions.

As a result of the research developments described herein, we can anticipate further significant enhancements in quality and productivity of oil crops over the coming decade. This will generate the technologies needed to support increasing plant oil production into the future, hopefully of sufficient magnitude to provide for significant use of renewable plant oils as replacements for fossil-derived industrial chemicals, without encroaching on the higher priority demand for food oils. Achievement of this goal will make a substantial contribution to moving to a sustainable carbon-neutral industrial society with lower emissions of carbon dioxide to the atmosphere, and reduced environmental impact as a result. In addition to the beneficial environmental effects, the agriculture will be a provider of high value added products that will substantially improve the economy of the rural societies.

## Abbreviations

ACP: acyl carrier protein
ALA: α-linolenic acid
DGAT: DAG acyltransferase
ER: endoplasmic reticulum
FAD: fatty acid desaturase
FAE: fatty acid elongase
FAR: fatty acid reductase
LPAAT: lysophosphatidic acid acyltransferase
LPC: lysophosphatidylcholine
LPCAT: lysophosphatidylcholine acyltransferases
PC: phosphatidylcholine
PDAT: phospholipid/DAG acyltransferase
PDCT: phospholipid/DAG cholinephosphotransferase
WS: wax synthase
